# Contribution of **E**pithelial **A**poptosis and **S**ubepithelial **I**mmune **R**esponses in *Campylobacter jejuni*-**I**nduced **B**arrier **D**isruption

**DOI:** 10.3389/fmicb.2020.00344

**Published:** 2020-03-06

**Authors:** Eduard Butkevych, Fábia Daniela Lobo de Sá, Praveen Kumar Nattramilarasu, Roland Bücker

**Affiliations:** Institute of Clinical Physiology/Nutritional Medicine, Medical Department, Division of Gastroenterology, Infectiology and Rheumatology, Charité-Universitätsmedizin Berlin, Berlin, Germany

**Keywords:** apoptosis, caspase, epithelial barrier, tumor necrosis factor alpha, *Campylobacter*, epithelial cell, immune cell co-culture, tight junction

## Abstract

*Campylobacter jejuni* is a widespread zoonotic pathogen and the leading bacterial cause of foodborne gastroenteritis in humans. Previous infection studies showed disruption of intercellular contacts, induction of epithelial apoptosis, and immune activation, all three contributing to intestinal barrier dysfunction leading to diarrhea. The present study aims to determine the impact of subepithelial immune cells on intestinal barrier dysfunction during *Campylobacter jejuni* infection and the underlying pathological mechanisms. Infection was performed in a co-culture of confluent monolayers of the human colon cell line HT-29/B6-GR/MR and THP-1 immune cells. Twenty-two hours after infection, transepithelial electrical resistance (TER) was decreased by 58 ± 6% compared to controls. The infection resulted in an increase in permeability for fluorescein (332 Da; 4.5-fold) and for FITC-dextran (4 kDa; 3.5-fold), respectively. In contrast, incubation of the co-culture with the pan-caspase inhibitor Q-VD-OPh during the infection resulted in a complete recovery of the decrease in TER and a normalization of flux values. Fluorescence microscopy showed apoptotic fragmentation in infected cell monolayers resulting in a 5-fold increase of the apoptotic ratio, accompanied by an increased caspase-3 cleavage and caspase-3/7 activity, which both were not present after Q-VD-OPh treatment. Western blot analysis revealed increased claudin-1 and claudin-2 protein expression. Inhibition of apoptosis induction did not normalize these tight junction changes. TNFα concentration was increased during the infection in the co-culture. In conclusion, *Campylobacter jejuni* infection and the consequent subepithelial immune activation cause intestinal barrier dysfunction mainly through caspase-3-dependent epithelial apoptosis. Concomitant tight junction changes were caspase-independent. Anti-apoptotic and immune-modulatory substances appear to be promising agents for treatment of campylobacteriosis.

## Introduction

Diarrheal disease is a major cause of morbidity and mortality worldwide. *Campylobacter jejuni* (*C. jejuni*) is a frequent commensal bacterium in poultry and wild birds and the leading cause of bacterial diarrhea in humans. As a zoonotic pathogen being highly contagious via the fecal-oral route, *C. jejuni* infection occurs by consumption of raw or undercooked meat, raw dairy products or contaminated water. The symptoms of the campylobacteriosis vary from fever, aches, and dizziness to severe manifestations with abdominal cramps and bloody diarrhea. The disease is self-limiting and antibiotic treatment is only recommended in chronic or severe cases. Nevertheless, *C. jejuni* infection result in very large health costs (Hoffmann et al., 2012; Tam and O’Brien, 2016) and can lead to complications such as post-infectious reactive arthritis and Guillain-Barré syndrome.

The pathogenesis of intestinal barrier dysfunction in the *C. jejuni* infection is not completely understood. During the infection, bacteria adhere to the mucus and transmigrate through the mucus layer and the epithelium (Backert et al., 2013) by invasion of enterocytes (Konkel et al., 1999; Song et al., 2004) or paracellularly with no changes in epithelial integrity (Boehm et al., 2012). Subsequent epithelial barrier impairment and activation of the innate inflammatory response was described *in vitro* in human cell cultures (Jones et al., 2003; Hu et al., 2006). These processes are also observed *in vivo* in *C. jejuni* patients (Spiller et al., 2000; Bücker et al., 2018) and in experimentally infected immune-deficient mice (Fox et al., 2004; Bereswill et al., 2011). In the pathogenesis of epithelial barrier dysfunction, apart from immune cell infiltration, tight junction changes, focal leaks and sodium malabsorption, the *C. jejuni*-induced epithelial cell death accompanies the pathological changes in the *C. jejuni*-infected mucosa.

In previous studies on the related bacteria *Arcobacter butzleri* or *Campylobacter concisus*, we were able to show that epithelial cell death and in particular apoptosis induction and not the compromised tight junction alone can lead to the epithelial barrier defect during infection (Bücker et al., 2009; Nielsen et al., 2011).

Two canonical pathways of apoptosis activation have been elucidated - the extrinsic pathway and the intrinsic pathway. Induction of these pathways results in activation of initiator caspases, leading to apoptosis commitment. The extrinsic pathway is triggered by ligand binding to the tumor necrosis factor (TNF) receptor superfamily members. The intrinsic pathway involves the release of caspase-activating factors by mitochondria in response to intracellular injuries such as DNA damage. Initiator caspases are then able to cleave pro−caspases and thus turn on downstream effector pro−caspases -3, -6, and -7, which in turn activate or inhibit target proteins, leading to apoptotic cell death (Delhalle et al., 2003). Apoptosis in the gut is associated with intestinal cell shedding – extrusion of enterocytes at the surface as a result of the migration from the base of the crypt to the top of the epithelium (Bullen et al., 2006). This process promotes a continuous turnover of the intestinal cells achieved without loss of intestinal barrier function.

Epithelial apoptosis is a physiological process, but if stimulated it can exceed the regenerative capacity of the mucosa and the epithelium cannot sustain a proper barrier function. Increased apoptosis causes (i) a reduction of the transepithelial resistance (TER), (ii) loss of water and electrolytes as well as (iii) increased permeability for macromolecules leaking into the lumen (leak-flux diarrhea) or (iv) increased antigen uptake from the lumen into the organism (leaky gut) (Bojarski et al., 2001). Enhanced antigen presentation to the submucosal immune cells, being part of the “leaky gut” phenomenon, reinforces the inflammation. This signifies that the disease enters a vicious circle, with the result that the intestinal tissue damage may rise to extremes.

We hypothesize induction of apoptosis as possible pathomechanism, induced indirectly by cytokines or together with direct *C. jejuni* effectors, affecting cellular viability and epithelial integrity. Although an increase of epithelial apoptosis in *C. jejuni*-infected tissue is evident, its mechanisms and impact on epithelial barrier function have not been elucidated yet and was not taken under consideration of the immune response in an *in vitro* model.

In the present study, we applied a recently described *C. jejuni* infection model in a co-culture of HT-29/B6-GR/MR epithelial and THP-1 immune cells to investigate the mechanisms leading to intestinal barrier disruption during the infection, such as epithelial cell death and tight junction changes, as well as the impact of subepithelial immune activation.

## Materials and Methods

### Co-culture of Human Epithelial Cells and Macrophage-Like Immune Cells

**FIGURE 1:**
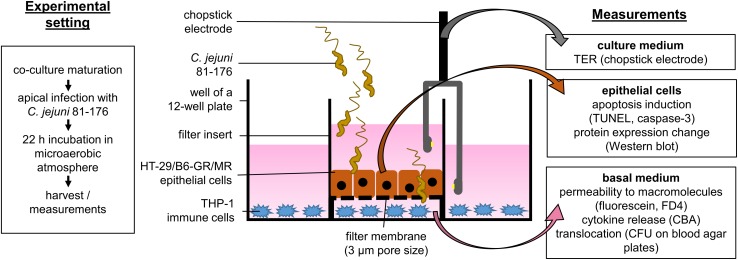
Schematic sideview on the co-culture infection model. Epithelial cells HT-29/B6-GR/MR grown on filter supports were placed in a 12-well plate containing stimulated THP-1 immune cells. The co-culture was inoculated with *C. jejuni* 81-176 reference stain from the apical side. The infection experiments were performed under microaerobic conditions for 22 h with or without the apoptosis inhibitor Q-VD-OPh. After 22 h electrophysiological measurements were performed to assess the epithelial barrier integrity. Epithelial cells and basal medium were then harvested. The samples were further processed to evaluate the effects of the infection as well as the pathological mechanisms of epithelial barrier dysfunction caused by *C. jejuni*.

We performed the infection experiments in a co-culture of HT-29/B6-GR/MR epithelial cells and THP-1 immune cells as recently described (Lobo de Sá et al., 2019) with the modification of the filter insert with larger pore size to allow bacterial translocation. Briefly, HT-29/B6-GR/MR cells (Bergann et al., 2011) were cultivated in 25 cm^2^ culture flasks for 7 days in RPMI 1640 culture medium (Sigma Aldrich, St. Louis, MO, United States) supplemented with 10% fetal calf serum (FCS; Gibco, Carlsbad, CA, United States), 1% penicillin/streptomycin (Corning, Wiesbaden, Germany), G418 (300 μg/ml; Invitrogen, Carlsbad, CA., United States) and hygromycin B (200 μg/ml; Biochrom GmbH, Berlin, Germany). For experimental use, cells were grown on 3 μm pore size Millicell PCF filters membranes (Merck Millipore, Billerica, MA, United States) at a density of 10^6^ cells cm^–2^ with a medium change every 2 days for 9 to 11 days till confluence. On the day of the experiment, the cells were washed three times and incubated for at least 1 h in antibiotic-free culture medium in the presence of 10% heat-inactivated FCS. THP-1 cells were incubated in 12-well plates with the antibiotic-free medium in the presence of 10% heat inactivated FCS and 100 nM phorbol 12-myristate 13-acetate (PMA; Sigma Aldrich, St. Louis, MO, United States; solved in DMSO). After 24 h the culture medium was removed, adhesion and differentiation state of THP-1 cells were controlled under a light microscope. The co-culture was started by placing the PCF filters with HT-29/B6-GR/MR cells into 12-well plates with adherent THP-1 immune cells at the bottom of the plate (Figure 1).

### Pharmacological Inhibitors

For the inhibition of apoptosis, we incubated the *C. jejuni* infected and not infected co-culture of HT-29/B6-GR/MR and THP-1 cells with 10 μM Q-VD-OPh hydrate ((3S)-5-(2,6-difluorophenoxy)-3-[[(2S)-3-methyl-1-oxo-2-[(2-quinolinylcarbonyl) amino]butyl]amino] -4-oxo-pentanoic acid hydrate, Calbiochem, San Diego, CA, United States) solved in DMSO (Sigma Aldrich, St. Louis, MO, United States). Staurosporine (Sigma Aldrich, St. Louis, MO, United States) was used for the induction of apoptosis at the concentration of 1 μM. The culture medium was supplemented with the given pharmacological inhibitors for at least 1 h before the infection and during the whole duration of the experiment.

### *C. jejuni* Infection of the Co-culture and Bacterial Transmigration

*Campylobacter jejuni* 81-167 reference strain was cultivated for 2 days on blood agar plates (Columbia Agar with Sheep Blood; Oxoid, Wesel, Germany) in an impermeable plastic container at 37°C with Oxoid CampyGen gas packs (Thermo Scientific, Waltham, MA, United States) to establish microaerobic conditions. For infection, we harvested bacterial colonies using an inoculation loop and resuspended them in the antibiotic-free cell-culture medium. After at least 2.5 h of further incubation in microaerobic conditions, the bacteria were gently centrifuged at 5000 × *g*, 10°C for 2 min, in favor of bacterial viability. The bacteria pellet was resuspended in Dulbecco’s phosphate buffered saline (DPBS; Sigma Aldrich, St. Louis, MO, United States) for quantification of the infection dose. The number of bacteria was estimated using optical density measurement and adjusted to OD_600_ = 2. The HT-29/B6-GR/MR cells in the co-culture setting were infected on the apical side with a multiplicity of infection (MOI) of 350 (Figure 1).

For quantification of bacterial transmigration, 25 μl of medium were removed from the basolateral compartment of the 12-well plates at the time points of 6, 12, and 24 h post-infection. Samples were diluted in 10-fold steps with antibiotic-free culture medium, sufficiently vortexed and incubated on the blood agar plates for 36 h. The number of colony-forming units (CFU) was counted and adjusted by the dilution coefficient to calculate the number of transmigrated bacteria.

### Cytometric Bead Array and Flow Cytometry

At the timepoint of 22 h after infection we collected the medium from basal compartment of the 12 well plate to analyze the secretion of cytokines (IL-1β, IL-2, IL-4, IL-6, IL-10, IL-17A, IFN-γ, and TNF-α) during the infection using a human cytometric bead array kit and manufacturers protocol (CBA; BD Biosciences human Th1, Th2, Th17 Kit, Flex Set IL-1β, Franklin Lakes, NJ, United States). Flow cytometric measurement were performed with FACS CantoII (BD Biosciences; Franklin Lakes, NJ, United States) and analyzed with FACP Array software v3.0 (BD Biosciences, Franklin Lakes, NJ, United States).

### Measurement of Transepithelial Electrical Resistance

The transepithelial electrical resistance (TER) was assessed before and 22 h after the infection. We performed the measurement with a chopstick electrode set (STX2, World Precision Instruments, Sarasota, FL, United States) and an epithelial volt-ohm meter (Institute of Clinical Physiology, Charité, Berlin). Electrodes were washed with 80% ethanol and phosphate buffered saline (PBS; Sigma Aldrich, St. Louis, MO, United States) in between the measurements. In pre-tests we have measured the TER of infected monolayers at multiple time points over the incubation period to determine the earliest onset of the barrier effect (22 h post-infection). For the experiments, TER was measured in the epithelial monolayers before infection, then the co-cultures were placed in microaerobic atmosphere in favor of the bacteria. The monolayers were measured again after the incubation period of 22 h.

### Transepithelial Permeability

For the measurement of the epithelial permeability 10 μl of fluorescein (332 Da, 100 mM, Fluorescein sodium salt, Sigma Aldrich, St. Louis, MO, United States) or fluorescein isothiocyanate-dextran solution (FITC-dextran, 4 kDa, 20 mM, Sigma Aldrich, St. Louis, MO, United States) dissolved in the culture medium was added to the apical side of the cell monolayer on PCF filter membranes placed in 12-well plates. The basal medium was removed at three subsequent time points every 15 (fluorescein) or 30 (FITC-dextran) minutes for the fluorescence measurement in a spectroflourometer (Infinite, Tecan GmbH, Mäennedorf, Switzerland). The standard for the fluorescent molecule concentration was determined in a dilution series. The permeability of the cell monolayer for the macromolecules was calculated from flux divided by concentration difference.

### Apoptosis Staining and Tight Junction Immunofluorescence

We analyzed the epithelial apoptotic rate using the TUNEL protocol (*In situ* Cell Death Detection Kit, Roche, Mannheim, Germany). Cells grown on filter supports were fixed with 2% paraformaldehyde for 30 min at a time point of 22 h after *C. jejuni* infection, thereafter permeabilized with 0.5% Triton X-100. Cell monolayers were incubated with TUNEL reagent at 37°C, repeatedly washed with blocking solution containing 5% goat serum in DPBS. 4′,6-Diamidino-2-phenylindole (DAPI) was applied as a nuclear counterstain. Apoptosis-positive cells were visualized with confocal laser scanning microscopy (Zeiss LSM780, Jena, Germany) and counted per high-power field.

For immunostaining of tight junction proteins, the epithelial monolayers were washed with PBS, permeabilized with 0.5% Triton X-100 (Sigma Aldrich, St. Louis, MO, United States) for 7 min, and blocked for 10 min with 1% goat serum. Then the cells were incubated with the primary antibodies anti-occludin (1:100; Invitrogen, Carlsbad, CA, United States) and anti-ZO-1 (1:100; BD Biosciences, Franklin Lakes, NJ, United States) for 1 h at room temperature. Afterward, the cells were washed and incubated with the secondary antibodies; anti-rabbit-Alexa-Fluor-488 and anti-mouse-Alexa-Fluor-594 for 1 h (1:500; Invitrogen, Carlsbad, CA, United States). Finally, the cells were washed with water and ethanol, and embedded in ProTaq Mount Fluor (Biocyc, Luckenwalde, Germany). The subcellular distribution of the tight junctions was analyzed by confocal laser scanning microscopy (Zeiss LSM780, Jena, Germany).

### Western Blot and Caspase Activity Analysis

Expression of tight junction proteins and caspase-3 cleavage during *C. jejuni* infection were investigated by Western blot analysis. Proteins were extracted from cell lysates 22 h post-infection. Cell-culture medium from the filter compartment containing extruded cells was centrifuged at 10000 × *g*, 4°C for 20 min. Supernatant was removed, the pellets were set aside on ice. Cell monolayers were treated with an ice-cold cell lysis buffer (10 mM Tris (pH 7.5), 150 mM NaCl, 0,5% Triton X-100, 1% SDS, complete protease inhibitor cocktail (Roche, Mannheim, Germany). Cells were scraped from filter supports carefully and added to the centrifuged cell pellet. Protein extraction and quantification, electrophoretic separation, western blotting and immunostaining were performed as previously described (Lobo de Sá et al., 2019). Nitrocellulose membranes were blocked with 1% PVP40 + 0.05% Tween20 for 2 h and incubated slewing in a box with primary antibodies anti-occludin, anti-claudin-2 (1:1000; Sigma Aldrich), anti-tricellulin, claudin-1, -4, -5, -8 (1:1000; Invitrogen, Carlsbad, CA, United States), and anti-caspase-3-cleaved (1:1000; Cell Signaling Technology, Denvers, MA, United States) overnight, and anti-β-actin (1:10000; Sigma Aldrich) over 4 h at 4°C. Afterward the membranes were washed with TBST buffer and incubated for 2 h at the room temperature with peroxidase conjugated secondary antibodies goat anti-rabbit IgG or goat anti-mouse IgG (Jackson ImmunoResearch, Ely, United Kingdom) prepared with 1% milk powder in TBST. Nitrocellulose membranes were then placed in the SuperSignal West Pico PLUS chemiluminescent peroxidase substrate (Thermo Scientific, Waltham, MA, United States) for 2 or 5 min for anti-β-actin and other antibodies, respectively. The chemiluminescence was measured using Fusion FX7 (Vilber Lourmat, Eberhardzell, Germany). ImageJ 1.52o quantification software was used for densitometric analysis. Signal intensity values were normalized by the loading control (β-actin) (Schneider et al., 2012). In parallel, the activity of caspase-3/7 was assessed using the Caspase-3/7 activity assay kit following manufacturer’s protocol (SensoLyte; AnaSpec, Fremont, CA, United States). Cell lysates were incubated with caspase-substrate Ac-DEVD-AFC and the fluorescence intensity of the fluorogenic indicator product was measured at Ex/Em = 380 nm/500 nm (Tecan, Mäennedorf, Switzerland).

### Statistical Analysis

All data are expressed as mean values ± standard error of the mean (SEM). Statistical analysis was performed using two-way unpaired Student’s *t*-test with Bonferroni-Holm adjustment for multiple comparison. Significance level was set at α = 0.05.

## Results

### Subepithelial Immune Cells Caused Epithelial Barrier Dysfunction at an Early Time Point of *C. jejuni* Infection by Means of Apoptosis Induction

**FIGURE 2:**
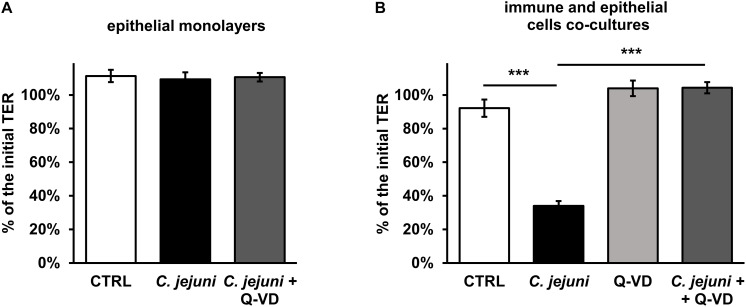
Epithelial barrier function during the first day of Campylobacter jejuni infection in the epithelial mono-culture and co-culture. TER values were assessed 22 h after infection for **(A)** HT-29/B6-GR/MR intestinal epithelial cell mono-culture infected with *C. jejuni* (MOI 350) with or without a broad-spectrum caspase inhibitor Q-VD-OPh (10 μM). *n* = 6. **(B)** The co-culture of HT-29/B6-GR/MR and THP-1 cells was infected with *C. jejuni* with or without the apoptosis inhibitor Q-VD-OPh. The TER values are presented as mean values ± SEM. *n* = 19–21, ***p < 0.001, unpaired Student’s *t*-test with Bonferroni-Holm correction for multiple comparisons.

*Campylobacter jejuni* is known to cause epithelial barrier dysfunction. Transepithelial electrical resistance (TER) served as a functional parameter to measure the integrity of the epithelial cell monolayer. As in prior studies, we confirmed that a barrier defect of *C. jejuni*-infected HT-29/B6 cell monolayer occurred only after 40 to 48 h after exposure to the cell mono-culture (Bücker et al., 2018) with 41 ± 4% of initial TER (*p* < 0.001, *n* = 7–8, data not shown). In line with this, at an earlier incubation time point of 22 h after infection, the TER did not drop (Figure 2A). Also, the apoptosis inhibitor Q-VD-OPh did not result in any significant alteration of the TER in the HT-29/B6-GR/MR cell culture on the first day of incubation. Since the mucosal immune activation in campylobacteriosis is a key feature of the disease, a co-culture of HT-29/B6/GR-MR and activated THP-1 immune cells was used for the infection assay. The contribution to the TER effect by the subepithelial THP-1 cells in an early incubation period was shown here in comparison to the unaffected mono-culture (Figures 2A,B). 22 h after exposure of the co-culture with *C. jejuni*, the infection caused a reduction in TER of 58 ± 6% (*p* < 0.001) compared to the controls (Figure 2B). Whereas, the co-culture incubated with the pan-caspase inhibitor Q-VD-OPh showed no changes in TER during the infection with *C. jejuni*. Surprisingly, the developing decrease in TER was completely blocked by Q-VD-OPh. Thus, the inhibition of apoptosis led to a full recovery of the barrier defect.

### Inhibition of Epithelial Apoptosis Normalized the Paracellular Permeability for Macromolecules in *C. jejuni* Infection

**FIGURE 3:**
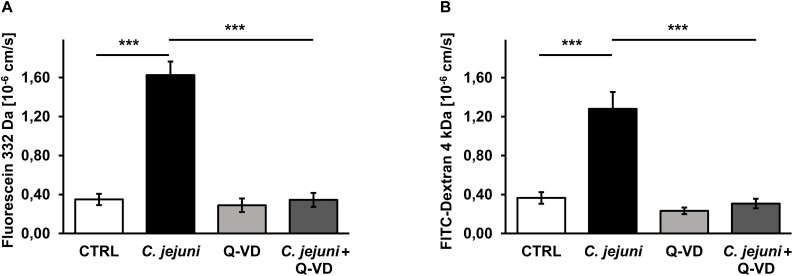
Epithelial permeability toward macromolecules in *C. jejuni*-infected co-cultures 22 h post-infection. Co-cultures of HT-29/B6-GR/MR and THP-1- cells were infected with *C. jejuni* with or without apoptosis inhibitor Q-VD-OPh. **(A)** Fluorescein flux and **(B)** FITC-Dextran flux across the epithelial monolayer were measured after 22 h of *C. jejuni* infection. The flux measurements were performed in the cell monolayer filter supports and are presented as mean values ± SEM, *n* = 6, ***p < 0.001, unpaired Student’s *t*-test with Bonferroni-Holm correction for multiple comparisons.

Besides TER measurements that rather reflect the ional permeability, we performed flux measurements of macromolecules in order to assess the barrier function for the paracellular leak pathway in our model. The permeability of the cell monolayer in the co-culture setting for small and mid-sized macromolecules changed during the *C. jejuni* infection. 22 h after infection, the fluorescein (332 Da) translocation from apical to the basal compartment increased 4.5-fold in infected samples compared to controls (*p* < 0.001), but remained steady under Q-VD-OPh treatment (Figure 3A). Similar to the fluorescein permeability, the FITC-dextran (4 kDa) translocation increased 3.5-fold during the infection (*p* < 0.001). Also, Q-VD-OPh incubation of the infected samples led to a full recovery of the FITC-dextran permeability (Figure 3B). The inhibition of apoptosis completely prevented the development of epithelial leaks and sufficiently sealed the epithelial barrier against the passage of macromolecules.

### *C. jejuni* Infection Changed the Expression of Tight Junction Proteins

**FIGURE 4:**
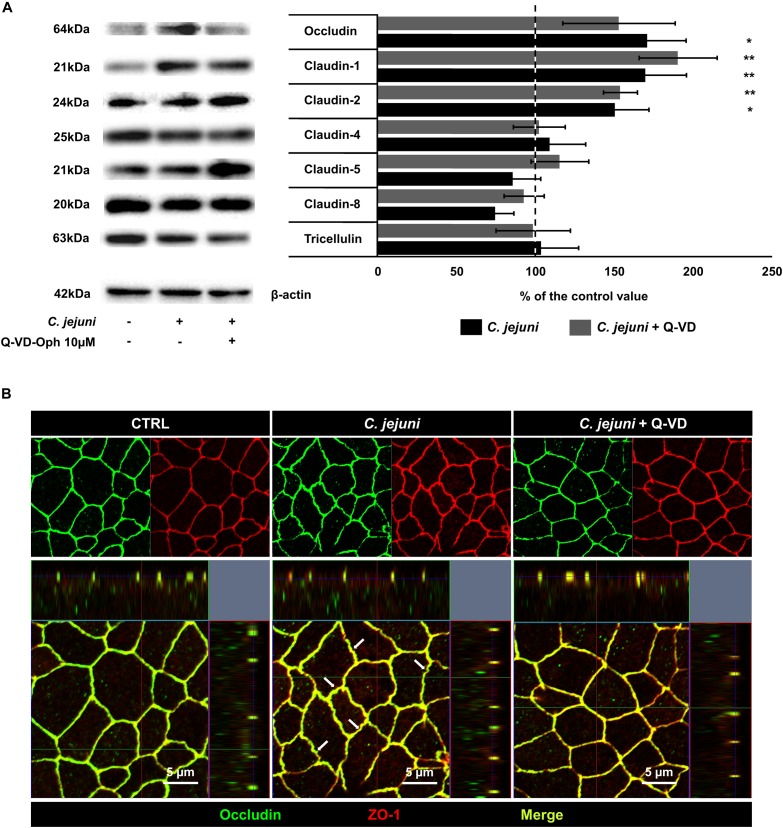
Expression of tight junction proteins in *C. jejuni*-infected co-cultures 22 h post-infection. Co-cultures of HT-29/B6-GR/MR and THP-1- cells were infected with *C. jejuni* with or without apoptosis inhibitor Q-VD-OPh. **(A)** Western blot was performed on the cell lysates 22 h after infection. Expression level of tight junction proteins was normalized with β-actin. Immunoblots were subjected to densitometric analysis. The dashed line represents control value set to 100%. Q-VD-OPh control treatment did not change the expression level of the described tight junction proteins compared to untreated controls (data not shown). Data are presented as mean values in percent of the control values, ± SEM. *n* = 7–9, *p < 0.05, **p < 0.01, unpaired Student’s *t*-test with Bonferroni-Holm adjustment for multiple comparisons. **(B)** The cell monolayers were analyzed by confocal microscopy after immunofluorescence staining. The micrographs show the localization of the tight junction proteins occludin (green) and zonula occludens protein-1; ZO-1 (red) at cell borders. The merge (yellow) image represents the co-localization of the tight junction proteins in a single plane of a *Z*-stack. White arrows indicate zigzag pattern of bicellular cell-cell contacts in *C. jejuni*-infected monolayers.

As molecular or cellular correlate to the barrier defect by *C. jejuni*, expression changes of tight junction proteins and/or epithelial cell damage (cell death) are thinkable. Therefore, we performed western blotting of treated epithelial cell monolayers. In the densitometric analysis of the Western blots, we discovered an almost 2-fold increase of an integral tight junction protein occludin (*p* < 0.05) and the barrier-forming claudin-1 protein (*p* < 0.01) expression. The claudin-1 effect was not diminished by apoptosis inhibition (Figure 4A). Cells incubated with Q-VD-OPh during the infection had similar increase in claudin-1 expression (*p* < 0.01) but only a change in occludin expression by trend. The channel-forming claudin-2 was also induced in HT-29/B6-GR/MR cells during the infection with (*p* < 0.01) and without (*p* < 0.05) concurrent apoptosis inhibition. Claudin-5 and claudin-8 show a reduction by trend that could develop an influence on the barrier integrity at a later time point of infection, whereas tricellulin and claudin-4 expression remained stable during the first hours of infection (Figure 4A). Tight junction expression changes were not prevented by apoptosis inhibition and presumably have a caspase-independent regulator. As the subcellular distribution of tight junction proteins can influence the epithelial barrier function, we analyzed immunofluorescent stainings of the treated monolayers. In confocal micrographs the intact co-localization of tight junction protein occludin together with zonula occludens protein-1 (ZO-1) was observed, without any re-distribution of tight junction protein signals to intracellular compartments in treated or infected cells (Figure 4B). At most a zigzag pattern of the bicellular junction became visible in infected monolayers, which might indicate the beginning of the following impact of *C. jejuni* on tight junction protein redistribution at later stages of infection.

### Epithelial Apoptosis in *C. jejuni* Infection Caused by Increased Caspase Activity

**FIGURE 5:**
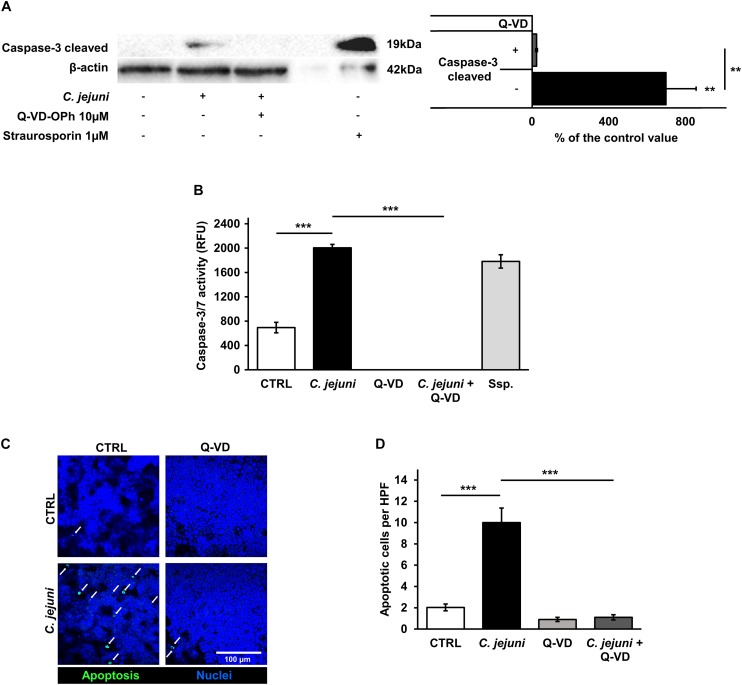
Effects of the *C. jejuni* infection on caspase activity. Co-cultures of HT-29/B6-GR/MR and THP-1 cells were infected with *C. jejuni* with or without apoptosis inhibitor Q-VD-OPh. **(A)** Caspase-3 cleavage in western blot after *C. jejuni* infection. Immunoblotting was performed on the cell lysates 22 h after infection. Staurosporine was used for the induction of apoptosis at the concentration of 1 μM as a positive control. Western blot densitometry represented in percent of the mean value in control samples. Western Blot intensity was normalized with β-actin level, *n* = 6, **p < 0.01. **(B)** Caspase-3/7 activity measured in a luminescense assay on cell lysates after 22 h of infection. Staurosporine (Ssp., 1 μM) incubated samples were used as positive controls. Data are represented in relative fluorescence units (RFU), *n* = 6, ***p < 0.001. **(C)** Apoptosis induction measured by TUNEL staining, showing DNA defragmentation in fluorescence microscopy. DAPI was applied as a nuclear counterstain. **(D)** Quantitative analysis of apoptotic cells in TUNEL staining (indicated by white arrows). Number of apoptosis positive cell nuclei was estimated in five high power fields per sample, containing approximately 1600 cells each. *n* = 6, ***p < 0.001, unpaired Student’s *t*-test with Bonferroni-Holm adjustment for multiple comparisons.

Although apoptosis can be initiated by different pathways, it ends up with a common final sequence of a few effector caspases. Therefore, we investigated the activation of caspase-3 in our infection model. The infection of the co-culture with *C. jejuni* resulted in an increased caspase-3 cleavage. In the densitometry analysis of Western blots, infected samples showed 9-fold increased band signal of the 19 kDa caspase-3 cleavage product (*p* < 0.01). This increase of the effector caspase was sufficiently blocked by the pan-caspase inhibitor Q-VD-OPh (*p* < 0.01) (Figure 5A). These changes correspond to the results of the caspase-3/7 activity assay. Here, a substantial rise of the effector caspase activity following the *C. jejuni* infection (*p* < 0.001) was measured with a completely inhibited activity, when incubated with Q-VD-OPh (*p* < 0.001) (Figure 5B). The effect of the changed number of apoptoses on the integrity of the cell monolayers was investigated after TUNEL staining by confocal laser scanning microscopy. *C. jejuni* infection led to 5-fold increase in the number of apoptotic cells in the cell culture. The increase in the apoptotic rate was prevented by incubation of the cell culture with the pan-caspase inhibitor Q-VD-OPh during the infection (*p* < 0.001) (Figures 5C,D). Thus, the predominant mechanism of the barrier defect in the early phase of infection was shown to be more caspase-dependent apoptosis induction than tight junction disruption.

### Cytokine Secretion Was Induced in *C. jejuni*-Infected Co-culture

**FIGURE 6:**
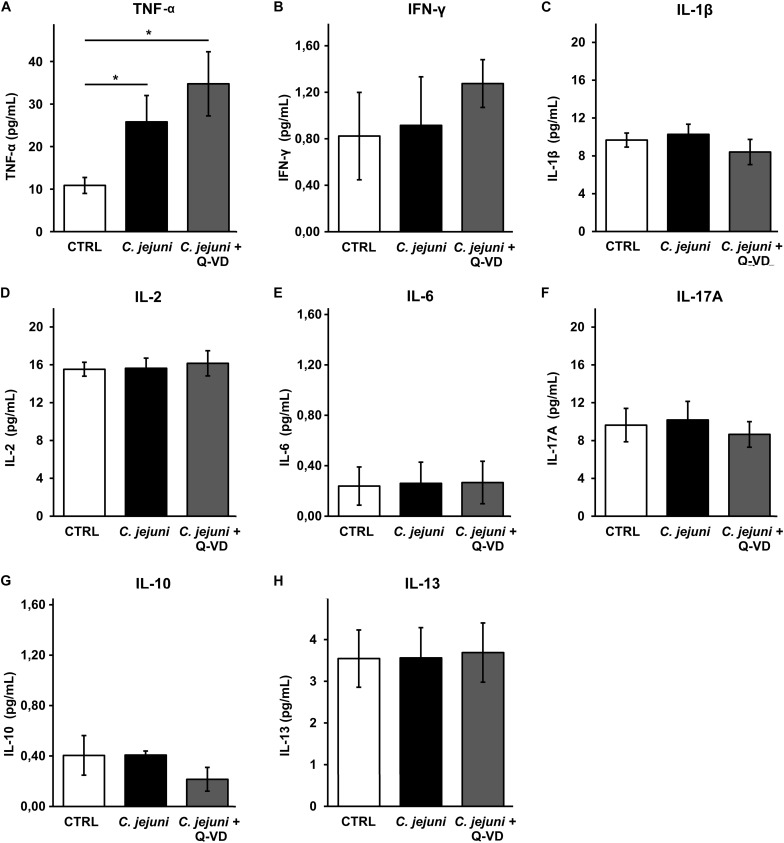
Immune activation during *C. jejuni* infection. Co-cultures of HT-29/B6-GR/MR and THP-1 cells were infected with *C. jejuni* with or without apoptosis inhibitor Q-VD-OPh. The secretion of the barrier relevant cytokines such as TNF-α, INF-γ, and IL-1β **(A–C)** as well as further interleukins **(D–H)** were assessed in the infected co-culture after 22 h. Cytokine concentration was measured in the culture medium from basal compartment. The concentrations are presented as mean values ± SEM, *n* = 6, *p < 0.05, unpaired Student’s *t*-test with Bonferroni-Holm adjustment for multiple comparisons.

Increased apoptosis induction can develop from direct bacterial contact and/or subepithelial cytokine release. To understand the mechanism of epithelial cell apoptosis we further investigated the secretion of cytokines from THP-1 cells in the infected co-culture. *C. jejuni* infection resulted in a 2.5-fold caspase-independent increase of TNFα concentration (*p* < 0.05). Immune activation was not affected by apoptosis inhibition with Q-VD-OPh (Figure 6A). Other pro-inflammatory cytokines such as IFN-γ, IL-1β, IL-2, IL-6 or IL-17 were not induced by *C. jejuni* or influenced by apoptosis inhibition after 22 h in the used infection setting (Figures 6B–F). Production of TH2 cytokines IL-10 and IL-13 also remained unchanged (Figures 6G,H). Therefore, the cell death receptor pathway of TNFα might play the major role in our experimental setup.

### *C. jejuni* Transmigration Across the Epithelium Was Not Limited by Apoptosis Inhibition

**FIGURE 7:**
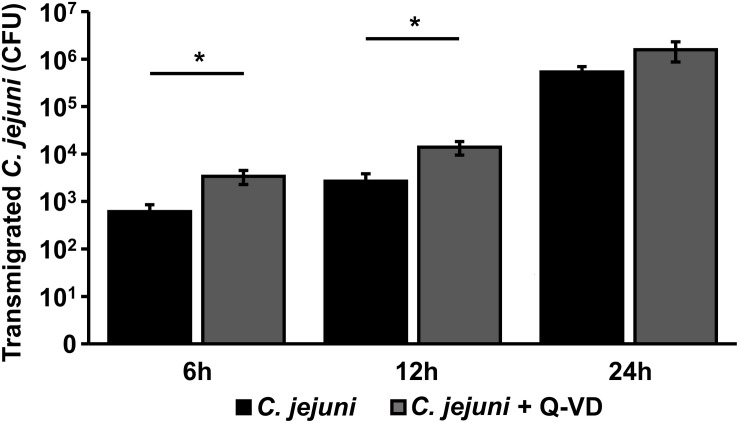
Bacterial transmigration through an epithelial monolayer during *C. jejuni* infection. Co-cultures of HT-29/B6-GR/MR and THP-1 cells were infected with *C. jejuni* with or without apoptosis inhibitor Q-VD-OPh. Transmigration of *C. jejuni* through the epithelial monolayer was assessed 6, 12, and 24 h after infection of the co-culture. The numbers of colony forming units (CFU) are presented as mean values ± SEM on a logarithmic scale, *n* = 12, *p < 0.05, unpaired Student’s *t*-test.

The translocation of *C. jejuni* and/or lipo**-**oligosaccharides (LOS) from the apical side through the epithelial monolayer, reaching the immune cells, is supposed as a pivotal step of the following aggravated outcome of the *C. jejuni* infection *in vivo*, leading to enhanced barrier disruption and again potentiated antigen influx into the subepithelium (leaky gut concept). In the transmigration experiment, we observed, that inhibition of apoptosis resulted not in a decrease, as expected, but in a 5.5-fold increase of bacterial translocation during the first 6 h of infection (*p* < 0.05, *n* = 6). After 12 h, the number of the bacteria, that reached basolateral compartment through the Q-VD-OPh incubated HT-29/B6-GR/MR cells, was only 3.5-times higher compared to the control infection in the co-culture (*p* < 0.05, *n* = 6) (Figure 7). At the time point of 24 h post-infection there was no significant difference between the two groups. Thus, the transmigration of *C. jejuni* was not limited by Q-VD-OPh, whereas the unrestricted passage of solutes and macromolecules could be diminished. Tight junction changes alone cannot explain the drop in resistance and increase in permeability for fluorescein and 4 kDa dextran. The increased apoptotic ratio in the monolayers seems to be the only barrier-relevant factor in the first phase of infection.

## Discussion

The perturbation of the intestinal barrier is considered to be a key mechanism for the development of *C. jejuni*-induced diarrhea. While apoptosis was hitherto insufficiently analyzed in the *C. jejuni*-infected epithelial cell culture, it is commonly referenced in *C. jejuni*-infected mice, apes and human tissues (Russell et al., 1993; Haag et al., 2012; Bücker et al., 2018). The role of apoptosis in epithelial barrier dysfunction was discussed but have never been investigated for the *C. jejuni* infection. Hence, we aimed on the effect of apoptosis induction on the barrier impairment caused by *C. jejuni* in an immune and epithelial cell co-culture approach to alleviate the diarrheal outcome of the disease. This novel method enabled us to study the interplay of *C. jejuni* with the epithelial monolayer and immune cells, reflecting an infection model for the delineation of the diarrheal symptoms of the infection.

### Q-VD-OPh as Inhibitor of *C. jejuni*-Triggered Barrier Dysfunction

For the first time we present a substance, the pan-caspase inhibitor Q-VD-OPh, which targets epithelial monolayers and completely inhibited the epithelial barrier dysfunction in *C. jejuni* infection. In the co-culture setting, it mitigated the decrease of the TER completely. No other compound was able to produce an outcome of this scale. In pre-tests, we targeted on the host cell signaling with the kinase inhibitors Y-27632 (PI3K) or ML-7 (MLCK) as well as blocking experiments on NF-κB activation (BAY 11-7082), but could not achieve any barrier-relevant protection effects (data not shown). In the literature, the improvement of barrier function was reported for use of vitamin D and PI3-kinase inhibitor LY294002 (Wine et al., 2008; Bücker et al., 2018). Their effects, ranging from mild to substantial, did not prevent from the loss of the barrier function, although *C. jejuni*-dependent activation of PI3K, ERK, p38, and NF-κB as well as impairment of actomyosin signaling were reported (Jin et al., 2003; Krause-Gruszczynska et al., 2007; Li et al., 2011). Recently published data on curcumin as a preventive drug against *C. jejuni*-induced barrier disruption showed a complete recovery of the barrier function of treated epithelial monolayers. Curcumin effects relied on immune regulation with a decreased level of cytokine production, protecting the enterocytes. In the same time frame, direct anti-apoptotic properties of curcumin were discussed (Lobo de Sá et al., 2019). Regarding the success of anti-apoptotic treatment with Q-VD-OPh, we consider apoptosis as a crucial pathomechanism for epithelial barrier dysfunction in the early stage of the *C. jejuni* infection.

### Inhibition of Apoptosis as Mechanism for Reducing Macromolecule Passage

Inhibition of apoptosis resulted in the recovery of the increased epithelial permeability toward macromolecules after infection to the level of control values. It is known that single-cell epithelial defects rapidly close by an actomyosin constriction “purse string” mechanism (Florian et al., 2002). As possible explanation for persistent barrier dysfunction, *C. jejuni* actively triggers signaling pathways to stimulate their own uptake by host cells using activation of the Rho GTPase, which in combination with induction of TNFα and INFγ pathways can lead to increased restitution time of single-cell lesions or increased numbers of cell extrusions (Günzel et al., 2006; Cróinín and Backert, 2012). Such mechanisms are also known for *Shigella*, *Salmonella*, and enterohaemorrhagic *Escherichia coli* during the initial stages of infection (Gudipaty and Rosenblatt, 2017). Increased epithelial permeability by single cell apoptosis facilitates unwanted loss of solutes and uptake of noxious agents (Gitter et al., 2000). One can conclude that apoptosis not only lead to barrier dysfunction in *C. jejuni* infection but can also be a part of the diarrheal mechanism and the cause of an excessive immune response, by increased antigen entry into the subepithelial compartment.

### No Downregulation of Barrier-Relevant Tight Junction Proteins

Tight junction proteins are the main determinants for sealing the paracellular pathway. Their impairment causes the leak flux mechanism by increased paracellular permeability (Barmeyer et al., 2017). Considering the TER reduction and increased macromolecular leak flux caused by *C. jejuni*, we investigated the expression of tight junction proteins. Surprisingly, no downregulation of barrier-relevant tight junction proteins was observed. While the increased expression of claudin-2, a tight junction protein forming a channel for small cations and water, could partially explain the decrease in TER, but it could not be responsible for the translocation of larger molecules. The contribution of claudin-2 upregulation under inflammatory conditions is described *in vitro* and *in vivo* (Heller et al., 2005; Zeissig et al., 2007; Luettig et al., 2015).

In our co-culture model the reduced TER and increased paracellular flux could not be explained by the expression change of occludin. Increased expression of occludin was reported to strengthen the barrier (McCarthy et al., 1996), but without a sealing effect in TER by occludin *per se*, since occludin knockout mice did not develop a barrier impairment (Schulzke et al., 2005). Here, claudins with sealing properties maintain the paracellular barrier. However, it is a matter of debate whether in an occludin knockout animal or cell model, other tight junction proteins can functionally compensate a loss of occludin (Saitou et al., 2000; Schulzke et al., 2005). Also, in a Caco-2 occludin knockout cell monolayer, the integrity of the epithelial barrier was intact, likewise in the wildtype Caco-2 cells, as indicated by a stable TER. Interestingly, in the Caco-2 model the cleavage of occludin by the secreted *C. jejuni* protease HtrA was shown, that facilitated the paracellular movement of the bacteria between the host cells (Harrer et al., 2019). Occludin was described as a molecular target of *C. jejuni*, as well as other pathogens and bacteria or viruses, leading to a direct or indirect disruption of occludin. In our experiments, confocal micrographs of the *C. jejuni*-infected monolayers revealed no protein redistribution of occludin to intracellular compartments and the occludin signal was clearly present in the tight junction domain. In the same time a zigzag pattern of cell-cell contacts was visible that points to morphological changes or the pre-stage for the following tight junction changes (re-distribution or scattering) in the further course of infection.

In general, upregulation of claudin-1 increases the epithelial resistance and decreases paracellular permeability to macromolecules (Inai et al., 1999). We also observed a claudin-1 upregulation in *C. jejuni* infection, the claudin-1 paradox, claudin-1 increase while the TER is decreased, as also shown before for *C. fetus* and *C. jejuni in vivo.* (Bücker et al., 2017, 2018; Lobo de Sá et al., 2019). The overall increase of claudin-1 was associated with retraction of the protein from tight junction strands and localization in the basolateral membrane and intracellular compartments during the infection (Bücker et al., 2018). Both claudin-1 and claudin-2 upregulation is shown to be mediated by TNFα signaling (Amasheh et al., 2010). The TNFα level was increased in our *C. jejuni* infection model. Claudin-1 level is known to be strongly increased in apoptosis inductor-treated HT-29/B6 cells (Bojarski et al., 2004), but was caspase independent in our infection model. Although we could show a change in the expression of the measured tight junction proteins, similar expression change was observed in infected monolayers after Q-VD-OPh treatment that showed sufficient barrier function in TER measurement and no increase of paracellular permeability for macromolecules. Therefore, tight junction changes alone cannot explain the disturbed barrier function caused by *C. jejuni* infection and its recovery. As an example of the intact tight junction meshwork pattern we showed occludin in co-localization with ZO-1 in controls, infected and Q-VD-OPh-treated monolayers in our co-culture setting. We propose that ongoing changes in tight junction protein expression and distribution, with impact on barrier function as reported (Bücker et al., 2018; Lobo de Sá et al., 2019) arise later on, with epithelial apoptosis being the main manifestation in the beginning of the infection, as shown here.

### Impaired Barrier Function Results From Induction of Epithelial Apoptosis

The increased epithelial apoptosis was caspase-3-dependent, mediated by *C. jejuni* infection, and the subsequent increase in TNFα. While the related pathogens *Campylobacter concisus* and *Arcobacter butzleri* directly initiate epithelial cell death (necrosis and apoptosis) that were partially barrier relevant with significant effects on TER and molecule marker fluxes (Bücker et al., 2009; Nielsen et al., 2011), we did not find such evidence for *C. jejuni* in the literature. Hence, we could show for the first time that epithelial apoptosis has a main effect on epithelial barrier function in *C. jejuni* infection, and in the early phase (first day of infection) it is the predominant reason for barrier impairment.

### Cytotoxic Mechanism Causing Epithelial Apoptosis

Regarding the inflammatory input, TNFα was the main cytokine increased during the infection. TNFα-induced barrier dysfunction was mostly caused by single−cell apoptotic events, increasing focal conductivity of the epithelium (Schulzke et al., 2006). Nevertheless, the immune response to the interaction with *C. jejuni* caused barrier impairment and induced overlapping effects of apoptosis and tight junction changes (Lobo de Sá et al., 2019). We proposed that *C. jejuni* possesses direct pro-apoptotic effectors. Previously, several bacterial proteins were associated with epithelial cell death. Cytolethal distending toxin prepared from *C. jejuni* was reported to cause cell cycle arrest and apoptosis in HeLa and epithelial Caco-2, or monocytic 28SC cells, while having no impact on T84 cells (Whitehouse et al., 1998; Kalischuk et al., 2007; Jain et al., 2009). The *Campylobacter* serine protease HtrA was shown as a virulence factor in mouse models with increased caspase-3 activity in both gnotobiotic IL-10-deficient mice and infant mice infected with *C. jejuni* wild type compared to *htrA*-deficient mutants (Heimesaat et al., 2014a, b; Schmidt et al., 2019). In both models, higher levels of pro-inflammatory cytokines such as TNFα, IL-6, and IFNγ in the colonic mucosa were measured in HtrA^+^ *C. jejuni* infection. Thus, indirect effects on the epithelial cell death are conceivable. Cytokines secretion, e.g., IL-13, IL-1β, IFN-γ, IL-10, IL-6, IL-17A, and IL-2, did not differ from control values in our co-culture, with an exception of TNFα. Moreover, gamma-glutamyl transpeptidase was found to inhibit epithelial cells proliferation in Caco-2 and AGS cell lines but was not responsible for cellular apoptosis (Floch et al., 2014). Hence, although no bacterial factor produced by *C. jejuni* was reported to induce defined apoptosis in intestinal epithelial cells, defined cytotoxicity was shown before. Interestingly, cytokine cocktails composed of IFNγ, TNFα, IL-13, and IL-1β, found to be the main barrier relevant cytokines in *C. jejuni*-infected human biopsies, substantially aggravated the *C. jejuni*-mediated epithelial defects (Bücker et al., 2018). Treatment with TNFα, a combination of TNFα and IFNγ, as well as solely IL-13 was shown to cause barrier dysfunction not only by tight junction alterations but also by the means of epithelial apoptosis in HT-29/B6 cells and rat colon (Grotjohann et al., 2000; Gitter et al., 2006; Heller et al., 2008). Therefore, we consider synergistic effects of cytokines and pathogen-host interaction described in previous studies (Rees et al., 2008; Bücker et al., 2018) as the leading mechanism to epithelial apoptotic events. Thus, the co-culture setting as an infection model is particularly advantageous to further investigate these mechanisms.

### Apoptosis as a Limiting Mechanism of *C. jejuni* Translocation

We assume that apoptosis inhibition not only tightens the barrier for solutes and macromolecules but also limits the translocation of *C. jejuni* via the paracellular route. The ability of *C. jejuni* to translocate across epithelial cell monolayer by paracellular and transcellular routes, is a crucial step of the infection. By reaching the subepithelial matrix, pathogen initiates the antigen presentation to the mucosal immune cells and reaches receptors located at the basolateral cell membrane of epithelial cells, like integrin receptors, which were shown to be necessary for cellular invasion (Backert et al., 2013). Surprisingly, transmigration of *C. jejuni* increased in Q-VD-OPh incubated samples. We suggest that the transcellular translocation capacity was increased with the higher number of viable epithelial cells. Additionally, the sealing of the focal leaks caused by apoptosis requires cytoskeletal transformation (Florian et al., 2002). Actin rearrangement and/or microtubule dynamics were also involved in the bacterial invasion process (Krause-Gruszczynska et al., 2007). Hence, this process might interfere after repair of apoptotic single cell lesions, resulting in impaired bacterial translocation.

The importance of epithelial cell death suggests the use of anti-apoptotic substances for prevention or treatment of the *C. jejuni* enteritis. Although medical use of caspase-inhibitors like Q-VD-OPh is unreasonable, substances with pronounced anti-apoptotic and immune-modulatory effect, e.g., vitamin D, curcumin and myrrh, have already been proven to their effectiveness in cell-culture or mouse models (Rosenthal et al., 2017; Bücker et al., 2018; Lobo de Sá et al., 2019; Mousavi et al., 2019). These compounds represent a potent alternative to the antibiotic treatment of *C. jejuni* infection.

## Conclusion

Caspase-dependent epithelial apoptosis, caused either by TNFα or by direct bacterial cytotoxicity, are the main mechanisms of the *C. jejuni*-induced barrier dysfunction in the early state of infection. Epithelial apoptosis caused leakage of macromolecules from apical to the subepithelial compartment but was not associated with tight junction changes or increased bacterial transmigration. Induction of apoptosis is a key mechanism for the development of leak flux diarrhea. These mechanisms provide further insight into new therapeutic approaches for the campylobacteriosis.

## Data Availability Statement

The raw data supporting the findings of this article will be made available by corresponding author, RB, or first author, EB, to any qualified researcher upon reasonable request.

## Author Contributions

EB and RB: conceptualization. EB, FL, and PN: data curation and Formal analysis. RB: funding acquisition, methodology, project administration, and supervision. EB: investigation and Writing the original draft. FL: resources. FL, PN, and RB: writing, review and editing.

## Conflict of Interest

The authors declare that the research was conducted in the absence of any commercial or financial relationships that could be construed as a potential conflict of interest.

## References

[B1] Amasheh M. Fromm A. Krug S. M. Amasheh S. Andres S. Zeitz M. (2010). TNFα-induced and berberine-antagonized tight junction barrier impairment via tyrosine kinase, Akt and NFκB signaling. *J. Cell Sci.* 123 4145–4155. 10.1242/jcs.070896 21062898

[B2] Backert S. Boehm M. Wessler S. Tegtmeyer N. (2013). Transmigration route of *Campylobacter jejuni* across polarized intestinal epithelial cells: Paracellular, transcellular or both? *Cell Commun. Signal.* 11:72. 10.1186/1478-811X-11-72 24079544 PMC3850506

[B3] Barmeyer C. Fromm M. Schulzke J. D. (2017). Active and passive involvement of claudins in the pathophysiology of intestinal inflammatory diseases. *Pflugers Arch. Eur. J. Physiol.* 469 15–26. 10.1007/s00424-016-1914-6 27904960

[B4] Bereswill S. Fischer A. Plickert R. Haag L. M. Otto B. Kühl A. A. (2011). Novel murine infection models provide deep insights into the “Ménage à trois” of *Campylobacter jejuni*, microbiota and host innate immunity. *PLoS One* 6:e0020953. 10.1371/journal.pone.0020953 21698299 PMC3115961

[B5] Bergann T. Fromm A. Borden S. A. Fromm M. Schulzke J. D. (2011). Glucocorticoid receptor is indispensable for physiological responses to aldosterone in epithelial Na+ channel induction via the mineralocorticoid receptor in a human colonic cell line. *Eur. J. Cell Biol.* 90 432–439. 10.1016/j.ejcb.2011.01.001 21354648

[B6] Boehm M. Hoy B. Rohde M. Tegtmeyer N. Bæk K. T. Oyarzabal O. A. (2012). Rapid paracellular transmigration of *Campylobacter jejuni* across polarized epithelial cells without affecting TER: role of proteolytic-active HtrA cleaving E-cadherin but not fibronectin. *Gut Pathog.* 4:3. 10.1186/1757-4749-4-3 22534208 PMC3413534

[B7] Bojarski C. Gitter A. H. Bendfeldt K. Mankertz J. Schmitz H. Wagner S. (2001). Permeability of human HT-29/B6 colonic epithelium as a function of apoptosis. *J. Physiol.* 535 541–552. 10.1111/j.1469-7793.2001.00541.x 11533143 PMC2278785

[B8] Bojarski C. Weiske J. Schöneberg T. Schröder W. Mankertz J. Schulzke J. D. (2004). The specific fates of tight junction proteins in apoptopic epithelial cells. *J. Cell Sci.* 117 2097–2107. 10.1242/jcs.01071 15054114

[B9] Bücker R. Krug S. M. Fromm A. Nielsen H. L. Fromm M. Nielsen H. (2017). *Campylobacter fetus* impairs barrier function in HT-29/B6 cells through focal tight junction alterations and leaks. *Ann. N. Y. Acad. Sci.* 1405 189–201. 10.1111/nyas.13406 28662272

[B10] Bücker R. Krug S. M. Moos V. Bojarski C. Schweiger M. R. Kerick M. (2018). *Campylobacter jejuni* impairs sodium transport and epithelial barrier function via cytokine release in human colon. *Mucosal. Immunol.* 11 474–485. 10.1038/mi.2017.66 28766554

[B11] Bücker R. Troeger H. Kleer J. Fromm M. Schulzke J.-D. (2009). *Arcobacter butzleri* induces barrier dysfunction in intestinal HT-29/B6 cells. *J. Infect. Dis.* 200 756–764. 10.1086/600868 19604116

[B12] Bullen T. F. Forrest S. Campbell F. Dodson A. R. Hershman M. J. Pritchard D. M. (2006). Characterization of epithelial cell shedding from human small intestine. *Lab. Investig.* 86 1052–1063. 10.1038/labinvest.3700464 16909128

[B13] Cróinín T. Backert S. (2012). Host epithelial cell invasion by *Campylobacter jejuni*: trigger or zipper mechanism? *Front. Cell. Infect. Microbiol.* 2:25. 10.3389/fcimb.2012.00025 22919617 PMC3417527

[B14] Delhalle S. Duvoix A. Schnekenburger M. Morceau F. Dicato M. Diederich M. (2003). “An introduction to the molecular mechanisms of *Apoptosis*,” in *Annals of the New York Academy of Sciences*, ed. Braaten D. (New York, NY: New York Academy of Sciences), 1–8. 10.1196/annals.1299.001 15033687

[B15] Floch P. Pey V. Castroviejo M. Dupuy J. W. Bonneu M. de la Guardia A. H. (2014). Role of *Campylobacter jejuni* gamma-glutamyl transpeptidase on epithelial cell apoptosis and lymphocyte proliferation. *Gut Pathog.* 6:20. 10.1186/1757-4749-6-20 24995041 PMC4080688

[B16] Florian P. Schöneberg T. Schulzke J. D. Fromm M. Gitter A. H. (2002). Single-cell epithelial defects close rapidly by an actinomyosin purse string mechanism with functional tight junctions. *J. Physiol.* 545 485–499. 10.1113/jphysiol.2002.031161 12456828 PMC2290693

[B17] Fox J. G. Rogers A. B. Whary M. T. Ge Z. Taylor N. S. Xu S. (2004). Gastroenteritis in NF-kappaB-deficient mice is produced with wild-type *Camplyobacter jejuni* but not with *C. jejuni* lacking cytolethal distending toxin despite persistent colonization with both strains. *Infect. Immun.* 72 1116–1125. 10.1128/iai.72.2.1116-1125.2004 14742559 PMC321575

[B18] Gitter A. H. Bendfeldt K. Schmitz H. Schulzke J.-D. Bentzel C. J. Fromm M. (2006). Epithelial barrier defects in HT-29/B6 colonic cell monolayers induced by tumor necrosis factor-α. *Ann. N. Y. Acad. Sci.* 915 193–203. 10.1111/j.1749-6632.2000.tb05242.x 11193576

[B19] Gitter A. H. Bendfeldt K. Schulzke J. D. Fromm M. (2000). Leaks in the epithelial barrier caused by spontaneous and TNF-α-induced single-cell apoptosis. *FASEB J.* 14 1749–1753. 10.1096/fj.99-0898com 10973924

[B20] Grotjohann I. Schmitz H. Fromm M. Schulzke J. D. (2000). Effect of TNF alpha and IFN gamma on epithelial barrier function in rat rectum in vitro. *Ann. N. Y. Acad. Sci.* 915 282–286. 10.1111/j.1749-6632.2000.tb05255.x 11193589

[B21] Gudipaty S. A. Rosenblatt J. (2017). Epithelial cell extrusion: pathways and pathologies. *Semin. Cell Dev. Biol.* 67 132–140. 10.1016/j.semcdb.2016.05.010 27212253 PMC5116298

[B22] Günzel D. Florian P. Richter J. F. Troeger H. Schulzke J. D. Fromm M. (2006). Restitution of single-cell defects in the mouse colon epithelium differs from that of cultured cells. *Am. J. Physiol. Regul. Integr. Comp. Physiol.* 290 R1496–R1507. 10.1152/ajpregu.00470.2005 16397094

[B23] Haag L. M. Fischer A. Otto B. Plickert R. Kühl A. A. Göbel U. B. (2012). *Campylobacter jejuni* induces acute enterocolitis in gnotobiotic IL-10-/- mice via toll-like-receptor-2 and -4 signaling. *PLoS One* 7:e0040761. 10.1371/journal.pone.0040761 22808254 PMC3393706

[B24] Harrer A. Bücker R. Boehm M. Zarzecka U. Tegtmeyer N. Sticht H. (2019). *Campylobacter jejuni* enters gut epithelial cells and impairs intestinal barrier function through cleavage of occludin by serine protease HtrA. *Gut Pathog.* 11:4. 10.1186/s13099-019-0283-z 30805031 PMC6373145

[B25] Heimesaat M. M. Alutis M. Grundmann U. Fischer A. Tegtmeyer N. Böhm M. (2014a). The role of serine protease HtrA in acute ulcerative enterocolitis and extra-intestinal immune responses during *Campylobacter jejuni* infection of gnotobiotic IL-10 deficient mice. *Front. Cell. Infect. Microbiol.* 4:77. 10.3389/fcimb.2014.00077 24959425 PMC4050650

[B26] Heimesaat M. M. Fischer A. Alutis M. Grundmann U. Boehm M. Tegtmeyer N. (2014b). The impact of serine protease HtrA in apoptosis, intestinal immune responses and extra-intestinal histopathology during *Campylobacter jejuni* infection of infant mice. *Gut Pathog.* 6:16. 10.1186/1757-4749-6-16 24883112 PMC4040118

[B27] Heller F. Florian P. Bojarski C. Richter J. Christ M. Hillenbrand B. (2005). Interleukin-13 is the key effector Th2 cytokine in ulcerative colitis that affects epithelial tight junctions, apoptosis, and cell restitution. *Gastroenterology* 129 550–564. 10.1016/j.gastro.2005.05.002 16083712

[B28] Heller F. Fromm A. Gitter A. H. Mankertz J. Schulzke J.-D. (2008). Epithelial apoptosis is a prominent feature of the epithelial barrier disturbance in intestinal inflammation: effect of pro-inflammatory interleukin-13 on epithelial cell function. *Mucosal Immunol.* 1 (Suppl. 1), S58–S61. 10.1038/mi.2008.46 19079233

[B29] Hoffmann S. Batz M. B. Morris J. G. (2012). Annual cost of illness and quality-adjusted life year losses in the united states due to 14 foodborne pathogens. *J. Food Prot.* 75 1292–1302. 10.4315/0362-028X.JFP-11-417 22980013

[B30] Hu L. Bray M. D. Osorio M. Kopecko D. J. (2006). *Campylobacter jejuni* induces maturation and cytokine production in human dendritic cells. *Infect. Immun.* 74 2697–2705. 10.1128/IAI.74.5.2697-2705.2006 16622206 PMC1459697

[B31] Inai T. Kobayashi J. Shibata Y. (1999). Claudin-1 contributes to the epithelial barrier function in MDCK cells. *Eur. J. Cell Biol.* 78 849–855. 10.1016/S0171-9335(99)80086-7 10669103

[B32] Jain D. Prasad K. N. Sinha S. Vishwakarma A. L. (2009). Cell cycle arrest & apoptosis of epithelial cell line by cytolethal distending toxin positive *Campylobacter jejuni*. *Indian J. Med. Res.* 129 418–423. 19535837

[B33] Jin S. Song Y. C. Emili A. Sherman P. M. Loong Chan V. (2003). JIpA of *Campylobacter jejuni* interacts with surface-exposed heat shock protein 90α and triggers signalling pathways leading to the activation of NF-κB and p38 MAP kinase in epithelial cells. *Cell. Microbiol.* 5 165–174. 10.1046/j.1462-5822.2003.00265.x 12614460

[B34] Jones M. A. Tötemeyer S. Maskell D. J. Bryant C. E. Barrow P. A. (2003). Induction of proinflammatory responses in the human monocytic cell line THP-1 by *Campylobacter jejuni*. *Infect. Immun.* 71 2626–2633. 10.1128/iai.71.5.2626-2633.2003 12704137 PMC153272

[B35] Kalischuk L. D. Inglis G. D. Buret A. G. (2007). Strain-dependent induction of epithelial cell oncosis by *Campylobacter jejuni* is correlated with invasion ability and is independent of cytolethal distending toxin. *Microbiology* 153 2952–2963. 10.1099/mic.0.2006/003962-0 17768238 PMC2884957

[B36] Konkel M. E. Kim B. J. Rivera-Amill V. Garvis S. G. (1999). Bacterial secreted proteins are required for the internaliztion of *Campylobacter jejuni* into cultured mammalian cells. *Mol. Microbiol.* 32 691–701. 10.1046/j.1365-2958.1999.01376.x 10361274

[B37] Krause-Gruszczynska M. Rohde M. Hartig R. Genth H. Schmidt G. Keo T. (2007). Role of the small Rho GTPases Rac1 and Cdc42 in host cell invasion of *Campylobacter jejuni*. *Cell. Microbiol.* 9 2431–2444. 10.1111/j.1462-5822.2007.00971.x 17521326

[B38] Li Y. P. Vegge C. S. Brøndsted L. Madsen M. Ingmer H. Bang D. D. (2011). *Campylobacter jejuni* induces an anti-inflammatory response in human intestinal epithelial cells through activation of phosphatidylinositol 3-kinase/Akt pathway. *Vet. Microbiol.* 148 75–83. 10.1016/j.vetmic.2010.08.009 20863633

[B39] Lobo de Sá F. D. Butkevych E. Nattramilarasu P. K. Fromm A. Mousavi S. Moos V. (2019). Curcumin mitigates immune-induced epithelial barrier dysfunction by *Campylobacter jejuni*. *Int. J. Mol. Sci.* 20:4830. 10.3390/ijms20194830 31569415 PMC6802366

[B40] Luettig J. Rosenthal R. Barmeyer C. Schulzke J. D. (2015). Claudin-2 as a mediator of leaky gut barrier during intestinal inflammation. *Tissue Barriers* 3:e977176. 10.4161/21688370.2014.977176 25838982 PMC4372021

[B41] McCarthy K. M. Skare I. B. Stankewich M. C. Furuse M. Tsukita S. Rogers R. A. (1996). Occludin is a functional component of the tight junction. *J. Cell Sci.* 109 2287–2298. 8886979 10.1242/jcs.109.9.2287

[B42] Mousavi S. Lobo de Sá F. D. Schulzke J.-D. Bücker R. Bereswill S. Heimesaat M. M. (2019). Vitamin D in acute campylobacteriosis-results from an intervention study applying a clinical *Campylobacter jejuni* induced Enterocolitis model. *Front. Immunol.* 10:2094. 10.3389/fimmu.2019.02094 31552040 PMC6735268

[B43] Nielsen H. L. Nielsen H. Ejlertsen T. Engberg J. Günzel D. Zeitz M. (2011). Oral and fecal *Campylobacter* concisus strains perturb barrier function by apoptosis induction in HT-29/B6 intestinal epithelial cells. *PLoS One* 6:e0023858. 10.1371/journal.pone.0023858 21887334 PMC3161070

[B44] Rees L. E. N. Cogan T. A. Dodson A. L. Birchall M. A. Bailey M. Humphrey T. J. (2008). *Campylobacter* and IFNγ interact to cause a rapid loss of epithelial barrier integrity. *Inflamm. Bowel Dis.* 14 303–309. 10.1002/ibd.20325 18050297

[B45] Rosenthal R. Luettig J. Hering N. A. Krug S. M. Albrecht U. Fromm M. (2017). Myrrh exerts barrier-stabilising and -protective effects in HT-29/B6 and Caco-2 intestinal epithelial cells. *Int. J. Colorectal Dis.* 32 623–634. 10.1007/s00384-016-2736-x 27981377

[B46] Russell R. G. O’Donnoghue M. Blake D. C. Zulty J. DeTolla L. J. (1993). Early colonic damage and invasion of *Campylobacter jejuni* in experimentally challenged infant *Macaca mulatta*. *J. Infect. Dis.* 168 210–215. 10.1093/infdis/168.1.210 8515112

[B47] Saitou M. Furuse M. Sasaki H. Schulzke J. D. Fromm M. Takano H. (2000). Complex phenotype of mice lacking occludin, a component of tight junction strands. *Mol. Biol. Cell* 11 4131–4142. 10.1091/mbc.11.12.4131 11102513 PMC15062

[B48] Schmidt A. M. Escher U. Mousavi S. Boehm M. Backert S. Bereswill S. (2019). Protease activity of *Campylobacter jejuni* HtrA modulates distinct intestinal and systemic immune responses in infected secondary abiotic IL-10 deficient mice. *Front. Cell. Infect. Microbiol.* 9:79. 10.3389/fcimb.2019.00079 30984628 PMC6449876

[B49] Schneider C. A. Rasband W. S. Eliceiri K. W. (2012). NIH Image to ImageJ: 25 years of image analysis. *Nat. Methods* 9 671–675. 10.1038/nmeth.2089 22930834 PMC5554542

[B50] Schulzke J. D. Bojarski C. Zeissig S. Heller F. Gitter A. H. Fromm M. (2006). “Disrupted barrier function through epithelial cell apoptosis,” in *Annals of the New York Academy of Sciences*, ed. Braaten D. (Malden, MA: Blackwell Publishing Inc), 288–299. 10.1196/annals.1326.027 17057208

[B51] Schulzke J. D. Gitter A. H. Mankertz J. Spiegel S. Seidler U. Amasheh S. (2005). Epithelial transport and barrier function in occludin-deficient mice. *Biochim. Biophys. Acta - Biomembr.* 1669 34–42. 10.1016/j.bbamem.2005.01.008 15842997

[B52] Song Y. C. Jin S. Louie H. Ng D. Lau R. Zhang Y. (2004). FlaC, a protein of *Campylobacter jejuni* TGH9011 (ATCC43431) secreted through the flagellar apparatus, binds epithelial cells and influences cell invasion. *Mol. Microbiol.* 53 541–553. 10.1111/j.1365-2958.2004.04175.x 15228533

[B53] Spiller R. C. Jenkins D. Thornley J. P. Hebden J. M. Wright T. Skinner M. (2000). Increased rectal mucosal enteroendocrine cells, T lymphocytes, and increased gut permeability following acute *Campylobacter enteritis* and in post-dysenteric irritable bowel syndrome. *Gut* 47 804–811. 10.1136/gut.47.6.804 11076879 PMC1728147

[B54] Tam C. C. O’Brien S. J. (2016). Economic cost of campylobacter, norovirus and rotavirus disease in the United Kingdom. *PLoS One* 11:e0138526. 10.1371/journal.pone.0138526 26828435 PMC4735491

[B55] Whitehouse C. A. Balbo P. B. Pesci E. C. Cottle D. L. Mirabito P. M. Pickett C. L. (1998). *Campylobacter jejuni* cytolethal distending toxin causes a G2-phase cell cycle block. *Infect. Immun.* 66 1934–1940. 10.1128/iai.66.5.1934-1940.1998 9573072 PMC108146

[B56] Wine E. Chan V. L. Sherman P. M. (2008). *Campylobacter jejuni* mediated disruption of polarized epithelial monolayers is cell-type specific, time dependent, and correlates with bacterial invasion. *Pediatr. Res.* 64 599–604. 10.1203/PDR.0b013e31818702b9 18679160

[B57] Zeissig S. Bürgel N. Günzel D. Richter J. Mankertz J. Wahnschaffe U. (2007). Changes in expression and distribution of claudin 2, 5 and 8 lead to discontinuous tight junctions and barrier dysfunction in active Crohn’s disease. *Gut* 56 61–72. 10.1136/gut.2006.094375 16822808 PMC1856677

